# Association between parental psychopathology and suicidal behavior among adult offspring: results from the cross-sectional South African Stress and Health survey

**DOI:** 10.1186/1471-244X-14-65

**Published:** 2014-03-04

**Authors:** Lukoye Atwoli, Matthew K Nock, David R Williams, Dan J Stein

**Affiliations:** 1 Department of Mental Health, School of Medicine, Moi University College of Health Sciences, Eldoret, Kenya; 2 Department of Psychiatry and Mental Health, University of Cape Town, Cape Town, South Africa; 3 Department of Psychology, Harvard University, Cambridge, MA, USA; 4 Department of Society, Human Development and Health, Harvard School of Public Health, Boston, MA, USA

**Keywords:** Suicide, Parental psychopathology, South Africa

## Abstract

**Background:**

Prior studies have demonstrated a link between parental psychopathology and offspring suicidal behavior. However, it remains unclear what aspects of suicidal behavior among adult offspring are predicted by specific parental mental disorders, especially in Africa. This study set out to investigate the association between parental psychopathology and suicidal behavior among their adult offspring in a South African general population sample.

**Method:**

Parental psychopathology and suicidal behavior in offspring were assessed using structured interviews among 4,315 respondents from across South Africa. The WHO CIDI was used to collect data on suicidal behavior, while the Family History Research Diagnostic Criteria Interview was used to assess prior parental psychopathology. Bivariate and multivariate survival models tested the associations between the type and number parental mental disorders (including suicide) and lifetime suicidal behavior in the offspring. Associations between a range of parental disorders and the onset of subsequent suicidal behavior (suicidal ideation, plans, and attempts) among adult offspring were tested.

**Results:**

The presence of parental psychopathology significantly increased the odds of suicidal behavior among their adult offspring. More specifically, parental panic disorder was associated with offspring suicidal ideation, while parental panic disorder, generalized anxiety disorder and suicide were significantly associated with offspring suicide attempts. Among those with suicidal ideation, none of the tested forms of parental psychopathology was associated with having suicide plans or attempts. There was a dose–response relationship between the number of parental disorders and odds of suicidal ideation.

**Conclusions:**

Parental psychopathology increases the odds of suicidal behavior among their adult offspring in the South African context, replicating results found in other regions. Specific parental disorders predicted the onset and persistence of suicidal ideation or attempts in their offspring. Further research into these associations is recommended in order to determine the mechanisms through which parent psychopathology increases the odds of suicidal behavior among offspring.

## Background

Suicide contributes significantly to the global burden of disease, and is recognized as one of the leading causes of mortality and years of life lost globally [1-3]. Interest in this area has resulted in a series of epidemiological studies on suicide and related risk factors, although most of this work originates from higher income countries [1,4,5]. Recent work from the South Africa Stress and Health (SASH) study suggested that rates of suicidal behavior in less developed countries in Africa may be comparable to findings in the more developed ones [6,7]. In the SASH study, the lifetime prevalence rates of suicidal ideation, suicide plans and suicide attempts were 9.1%, 3.8% and 2.9% respectively [6].

Despite the growing body of research on suicide, relatively little is known about the predictors of suicidal ideation and attempts in the African setting. Even less is known about what factors lead those with suicidal ideation to go on to make suicide attempts. Prior studies in non-African settings have shown that suicidal behavior tends to run in families, and it has been suggested that some aspects of this are mediated at least in part by mental illness among relatives [8-13]. In particular, it has been shown that whereas suicidal ideation may be mediated by family history of mental illness, suicide attempts are more likely to be associated with family history of impulsive-aggressive behavior and mood disorders [9,10,12,14-17].

Previous work from the World Mental Health Survey Initiative has established that a wide range of parental mental disorders are associated with an increased the risk of suicidal ideation among the offspring [18]. It has been demonstrated that parental depression and generalized anxiety disorder predict onset and persistence of suicide plans, whereas parental antisocial personality disorder and anxiety disorders predict onset and persistence of suicidal attempts among those with suicidal ideation [18].

Apart from the World Mental Health Survey, previous population-based studies on parental psychopathology and the risk of suicide in the offspring have been carried out mainly in high income countries. Studies from Denmark and Sweden have shown that completed suicide and psychiatric illness in parents and other relatives are risk factors for offspring suicide, and that the effect of family suicide history is independent of the familial cluster of mental disorders [19-21]. Additionally, Stenager and Qin [22] showed a greater effect of maternal mental illness on offspring suicide, as well as the fact that the elevated risk associated with parental psychiatric history was greater in females than in males, and tended to be more prominent during the first few years after admission of a parent.

In one of the few studies from Low and Middle Income Countries, a Nigerian study [23] found that parental panic disorder and substance abuse were associated with suicidal ideation in offspring, but only parental panic disorder was associated with suicide attempts. In that study, parental panic disorder was also reported to predict the onset and persistence of suicidal ideation and attempts, and also which persons with suicidal ideation go on to make a suicide attempt. The findings from this Nigerian study suggest that the range of parental mental disorders associated with offspring suicidal behavior would be narrower in the African setting than what has been reported in higher income countries.

The current analysis of the South African Stress and Health Study (SASH) [24] data sought to explore the relationship between a range of parental psychopathology and the occurrence of suicidal ideation and attempts in the offspring in a South African general population sample. The primary hypothesis was that parental disorders such as major depression would predict suicidal ideation while anxiety and antisocial personality disorder would predict offspring suicide attempts as has been suggested by previous studies [18,25,26]. We also intended to explore the role of parental panic disorder in offspring suicidal ideation, as suggested by the study in Nigeria [23].

Secondly, the study set out to further test the hypothesis that among people with suicidal ideation, those reporting parental antisocial personality disorder and anxiety disorders would have a higher risk of developing suicide plans and attempts. A number of previous studies have demonstrated such a link, but little has been done in Africa or in other low and middle income countries [10,11,17].

The present study therefore uses a larger sample based on the population of an African country in order to determine whether previous findings can be generalized to low and middle income countries.

## Method

The South African Stress and Health (SASH) study [24] was carried out as part of the World Mental Health Survey [27] to determine the prevalence rate and risk factors for mental disorders in South Africa. The survey was conducted between January 2002 and June 2004. The rationale and survey methods have been described in previous publications [24,28] and are briefly summarized here.

The SASH protocol was reviewed and approved by the Human Subjects Research Ethics Committees of the University of Michigan, Harvard Medical School and the Medical University of South Africa (MEDUNSA) [24].

The study population comprised adult South Africans residing both in households and hostels. Individuals living in institutions such as hospitals, prisons, mental health institutions and military bases were excluded from the study. A multi-stage area probability sample design was used to select the sample. In the first stage, a stratified probability sample of primary sampling areas was selected. These areas are roughly equivalent to counties in the US or the UK, and the size was based on the 2001 South African Census of Enumeration Areas (EAs). The EAs were sampled with probabilities proportionate to their population size. In the second stage, a random sample of 5 households was selected within each EA. The third stage consisted of a random sub-selection of a single adult respondent in each selected household. The study achieved an overall response rate was 85%, resulting in a final sample consisting of 4315 respondents.

The data collection proceeded province by province with a cohort of 40–60 interviewers in each province. All SASH interviewers were trained on consenting and administration of the study instruments in centralized group sessions lasting 1 week. The interviews were conducted face to face in one of six different languages spoken by most South Africans: English, Afrikaans, Zulu, Xhosa, Northern Sotho, and Tswana. Respondent interviews lasted approximately three and a half hours, and some of them took more than one visit to complete. Written informed consent was obtained from all the participants before the interviews were conducted.

### Measures

#### Sociodemographic variables

Sociodemographic variables included in this analysis were gender, age, marital status and education. Age consisted of four categories (in years): 18–29, 30–44, 45–59, and 60 or older. Marital status was categorized into three groups: married, previously married and never married. Education was classified depending on number of years of formal schooling into four categories: Low (0–1 year), low-average (2–7 years), high-average (8–12 years) and high (13 or more years).

#### Suicidal behavior

Suicidal behavior was assessed using the Suicidality Module of the World Health Organization (WHO) Composite International Diagnostic Interview (CIDI) Version 3.0, a fully structured diagnostic interview administered by trained lay interviewers [29]. This module includes an assessment of the lifetime occurrence and age-of-onset of suicidal ideation, plans, and attempts. Good inter-rater reliability, test–retest reliability, and validity have been found in earlier versions of the CIDI [30], while good validity of CIDI diagnoses compared to diagnoses based on blinded clinical reappraisal interviews have been found in WMH methodological studies [31].

As described previously [28], the translation of the English version of the CIDI into the five other languages used in the SASH study was carried out according to WHO recommendations of iterative back-translation conducted by panels of bilingual and multilingual experts. Discrepancies found in the back-translation were resolved by consensus of an expert panel.

In order to examine relations between parental psychopathology and a continuum of suicidal behaviors, we considered four dated lifetime history outcomes in a series of nested survival analyses: (i) suicidal ideation in the total sample; (ii) suicide attempt in the total sample; (iii) suicide plan among respondents with ideation; (iv) suicide attempt among respondents with ideation.

#### Parental psychopathology

Parental psychopathology was assessed using the Family History Research Diagnostic Criteria Interview [32] and its expansion [33]. In the current study we examined respondents’ report of five different forms of possible lifetime psychopathology among their parent(s) during their childhood: major depression, panic disorder, generalized anxiety disorder, substance dependence, and antisocial personality disorder, as well as parental suicide attempt or suicide death. Although the instrument measures more than these five areas of psychopathology, they were selected due to indications from previous research that they were the main factors associated with offspring suicidal behavior. Since this data was collected as part of the larger SASH study, it was important to focus on areas that were thought to be important in order to minimize the length of the interview while collecting all the required information.

This instrument has been found to have relatively good sensitivity, although there is a risk of under-reporting of familial psychopathology [32]. However it is useful in community surveys where a family study method would be very difficult to implement, including in situations where some family members are deceased.

### Data analysis

In order to account for the stratified multi-stage sample design, the data were weighted to adjust for differential probability of selection within households as a function of household size and clustering of the data, and for differential non-response. A post-stratification weight was also used to make the sample distribution comparable to the population distribution in the 2001 South African Census for age, sex, and province.

The prevalence of parental psychopathology among respondents with each of the four suicidal outcomes was estimated using cross-tabulations. Predictive associations between parental psychopathology and subsequent suicidal behaviors were estimated using discrete-time survival models with person-year as the unit of analysis [34]. Survival coefficients were exponentiated to generate odds-ratios (ORs) and their standard errors for ease of interpretation.

The associations between parental psychopathology and suicidal behavior were estimated in a series of survival models that were bivariate (in which each type of parental psychopathology was considered individually) as well as multivariate (in which all parental disorders were considered simultaneously) in predicting each suicidal behavior. Further models testing whether a greater number of parental disorders are associated with an increased risk of each suicidal behavior were estimated. All models controlled for age, gender, marital status and education of the respondents.

In all analyses, standard errors of prevalence estimates and survival coefficients were estimated with the Taylor series method [35] using SUDAAN software [36] to adjust for the weighting and clustering of the SASH sample design. Multivariate significance was evaluated with Wald *χ*^2^ tests based on design-corrected coefficient variance–covariance matrices. All significance tests were evaluated using .05 level two-sided tests.

## Results

The demographic characteristics of this sample have been described previously [37] but briefly, the sample was largely female (58.6%) and Black (79.7%), although other racial groups are represented (10.4% Colored; 7.2% White; 2.7% Indian/Asian). Further, one-half was married, while most were unemployed (69.2%), had less than 12 years of education (62.7%), and lived in urban areas (59.7%).

Out of the 4315 participants, 1.5% (n = 65) reported parental major depressive disorder, while 6.4% (n = 275) reported parental panic disorder, 1.4% (n = 62) parental generalized anxiety disorder, 3.8% (n = 162) parental substance use disorder, and 3.4% (n = 148) parental antisocial personality disorder. Parental suicide was reported by 1.6% (n = 71) of the participants.

As shown in Table 1, the lifetime prevalence rates of suicidal ideation (n = 394), suicide plans (n = 171) and suicide attempts (n = 140) were 9.1% (SE 0.7), 3.8% (SE 0.4) and 2.9% (SE 0.3) respectively.

**Table 1:** Prevalence of parental psychopathology among suicidality outcomes

**Parental disorder (n)**		**In the total sample**				**Among those with suicidal Ideation**			
		**%**^**1**^**(SE) with parent disorder among:**		**%**^**1**^**(SE) with parent disorder among:**		**%**^**1**^**(SE) with parent disorder among:**		**%**^**1**^**(SE) with parent disorder among:**	
		**Attempt**	**No attempt**	**Ideation**	**No ideation**	**Plan**	**No plan**	**Attempt**	**No attempt**
Depression (65)		0.9 (0.7)	1.5 (0.3)	1.6 (0.7)	1.5 (0.3)	1.0 (0.6)	2.7 (1.3)	0.9 (0.7)	2.5 (1.2)
Panic (275)		14.2 (3.9)	6.1 (0.8)	9.1 (1.7)	6.0 (0.8)	14.5 (3.4)	6.5 (1.8)	14.2 (3.9)	6.6 (1.7)
GAD (62)		2.3 (1.3)	1.4 (0.3)	3.0 (0.9)	1.3 (0.3)	3.1 (1.6)	3.4 (1.8)	2.3 (1.3)	2.2 (1.1)
Substance abuse (162)		8.0 (2.2)	3.6 (0.4)	5.7 (1.1)	3.5 (0.4)	5.0 (1.7)	7.4 (2.4)	8.0 (2.2)	5.6 (1.8)
Anti-social personality disorder (148)		7.6 (2.1)	3.3 (0.4)	5.4 (1.1)	3.2 (0.4)	6.5 (1.4)	7.7 (3.0)	7.6 (2.1)	6.4 (2.4)
Suicide (Same-sex parent) (71)		3.0 (1.8)	1.6 (0.3)	4.2 (1.1)	1.4 (0.3)	2.5 (1.6)	7.3 (3.8)	3.0 (1.8)	7.1 (3.8)
Number of parental disorders	1	18.4 (3.4)	9.3 (0.7)	15.5 (2.0)	9.1 (0.7)	17.1 (3.5)	19.8 (5.2)	18.4 (3.4)	17.2 (4.9)
	2+	8.1 (2.6)	3.2 (0.4)	5.5 (1.1)	3.1 (0.4)	6.6 (1.7)	6.3 (2.2)	8.1 (2.6)	5.1 (1.6)
(*N*^2^,%)		(140, 2.9%)	(4175, 97.1%)	(394, 9.1%)	(3921, 90.9%)	(171, 43.4%)	(223, 56.6%)	(140, 35.5%)	(254, 64.5%)

^1^Percentage (%) represents the percentage of people with the parent disorder among the cases with the outcome variable indicated in the column header. Prevalence estimates are from personyear data. For example: the first cell shows that 0.9% of those who attempted suicide had at least one parent with MDE.

^2^*N* represents the number of cases with the outcome variable, followed by the applicable percentage.

In this study, 21% of participants with suicidal ideation reported parental psychopathology, compared to 12.2% of those without suicidal ideation. Over 26% of those who attempted suicide also reported at least one parental mental disorder, compared to 12.5% of those without suicide attempts. Among those with suicidal ideation, 23.7% of those who proceeded to develop a suicide plan reported at least one parental mental disorder while those who did not have a plan reported 26.1% parental psychopathology.

Table 1 shows these prevalence rates aggregated by type and number of parental disorders. Respondents with suicidal behavior reported higher rates of all the tested types of parental psychopathology except major depression.

Preliminary bivariate survival models were estimated for the association of each parental disorder with suicidal behavior in the respondent. The models tested the effect of parental psychopathology in either parent alone and in both parents. Among all the factors tested, only suicide in a parent of the same sex as the respondent predicted respondent suicidal ideation. As a result, all subsequent analyses tested the effects of psychopathology in either parent, except in the case of parental suicide where only suicide of a parent of the same sex as the respondent was considered.

As shown in Table 2, bivariate survival models demonstrated that parental panic disorder (OR = 2.5), substance abuse (OR = 2.1) and antisocial personality disorder (OR = 2.3) were associated with suicide attempts in respondents. In those with suicidal ideation, no type of parental psychopathology was associated with offspring suicide plans or attempts.

**Table 2:** **Bivariate associations between parental psychopathology and respondent lifetime suicidal behavior**^**1**^

	**In the total sample**		**Among those with suicidal ideation**	
	**Ideation**	**Attempts**	**Plans**	**Attempts**
	**OR (95% CI)**	**OR (95% CI)**	**OR (95% CI)**	**OR (95% CI)**
Depression	1.0 (0.5-2.2)	0.6 (0.1-2.4)	0.3 (0.0-1.7)	1.0 (0.1-8.1)
Panic	1.6* (1.1-2.3)*	2.5* (1.4-4.6)*	2.0 (0.9-4.2)	1.6 (0.7-3.9)
GAD	2.4* (1.4-4.0)*	1.8 (0.5-6.1)	1.1 (0.2-5.8)	1.2 (0.3-4.6)
Substance abuse	1.6* (1.0-2.4)*	2.1* (1.1-4.1)*	0.5 (0.2-1.5)	2.1 (0.8-5.5)
Anti-social personality disorder	1.6* (1.1-2.4)*	2.3* (1.2-4.3)*	0.8 (0.3-1.9)	1.0 (0.3-3.3)
Suicide (same-sex parent)	3.0* (1.7-5.5)*	1.9 (0.5-6.5)	0.4 (0.1-1.7)	0.4 (0.0-8.1)

*Significant at the .05 level, two-sided test.

^1^Each row represents a bivariate model. Models control for person-year (1–5 intervals) and demographic factors (age, sex, education level, and marital status).

When a multivariate model was estimated in which each form of parental psychopathology was entered simultaneously, parental generalized anxiety disorder (OR = 2.4) and suicide (OR = 2.7) remained significantly associated with suicidal ideation among respondents, whereas parental panic disorder (OR = 2.5) was associated with respondent suicide attempts. As illustrated in Table 3, among those with suicidal ideation, parental panic disorder (OR = 2.5, 95% CI 1.2-5.2) was significantly associated with having suicide plans, but no form of parental psychopathology was significantly associated with suicide attempts among ideators.

**Table 3:** **Multivariate associations between parental psychopathology and respondent lifetime suicidal behavior**^**1**^

	**In the total sample**		**Among those with suicidal ideation**	
	**Ideation**	**Attempts**	**Plans**	**Attempts**
	**OR (95% CI)**	**OR (95% CI)**	**OR (95% CI)**	**OR (95% CI)**
Depression	0.4 (0.1-1.2)	0.2 (0.0-1.1)	0.2 (0.0-2.1)	0.6 (0.0-10.5)
Panic	1.5 (1.0-2.2)	2.5* (1.3-4.7)*	2.5* (1.2-5.2)*	1.5 (0.6-4.1)
GAD	2.4* (1.2-4.8)*	1.5 (0.4-6.3)	2.0 (0.4-10.5)	0.8 (0.2-4.1)
Substance abuse	1.3 (0.8-2.1)	1.6 (0.8-3.4)	0.5 (0.1-1.6)	2.1 (0.7-6.9)
Anti-Social personality disorder	1.3 (0.9-2.1)	1.8 (0.9-3.8)	1.0 (0.3-3.1)	0.7 (0.2-2.5)
Suicide (Same-sex parent)	2.7* (1.5-4.9)*	1.5 (0.4-5.0)	0.4 (0.1-1.7)	0.4 (0.0-7.6)

*Significant at the .05 level, two-sided test.

^1^Models control for person-year (1–5 intervals) and demographic factors (age, sex, education level, and marital status).

The association between the number of parental disorders and respondent suicidal behavior was estimated using a multivariate model in which only substantive predictors were used as dummy variables for the number of parental disorders. Due to the fact that very few respondents had parents with more than two disorders, only two categories were created for number of disorders, those with one disorder, and those with two or more disorders.

Compared to those with no parental disorders, respondents reporting one parental disorder had more than double the odds of suicidal ideation (OR = 2.4), and those with two or more parental disorders had nearly a three-fold increase in the odds of ideation (OR = 2.8). For suicide attempts, the magnitude of the association was similar between those with one parental disorder (OR = 1.9) compared to those without, and those with two or more parental disorders (OR = 1.9). Among those with suicidal ideation, there was no association between number of parental disorders and respondent suicide plans or attempts. This is summarized in Table 4.

**Table 4:** **Multivariate associations between number of parental disorders and respondent suicidal behavior**^**1**^

	**In the total sample**		**Among those with suicidal ideation**	
	**Ideation**	**Attempts**	**Plans**	**Attempts**
**Number of parental disorders**	**OR (95% CI)**	**OR (95% CI)**	**OR (95% CI)**	**OR (95% CI)**
1	2.4* (1.5-3.8)*	1.9* (1.4-2.6)*	0.9 (0.4-2.0)	1.1 (0.4-3.0)
2+	2.8* (1.3-5.8)*	1.9* (1.3-2.8)*	1.0 (0.4-2.5)	2.0 (0.8-4.8)

*Significant at the .05 level, two-sided test.

^1^ Models control for person-years (1–5 intervals) and demographic factors (age, sex, education level, and marital status).

In a final multivariate model including both type and number of parental disorders, parental panic disorder continued to predict respondent suicidal ideation (OR = 2.5), whereas parental panic disorder (OR = 1.6), generalized anxiety disorder (OR = 2.8) and suicide (OR = 3.0) predicted respondent suicide attempts. As shown in Table 5, however, in those with suicidal ideation there was no statistically significant association between any parental disorder and suicide plan or attempt.

**Table 5:** **Multivariate model of the associations between type and number of parental disorders and Respondent suicidal behavior**^**1**^

	**In the total sample**		**Among those with suicidal Ideation**	
	**Ideation**	**Attempts**	**Plans**	**Attempts**
	**OR (95% CI)**	**OR (95% CI)**	**OR (95% CI)**	**OR (95% CI)**
Depression	0.2 (0.0-1.2)	0.4 (0.1-1.5)	0.1 (0.0-1.3)	0.6 (0.0-9.8)
Panic	2.5 (1.4-4.7)*	1.6 (1.1-2.4)*	2.1 (0.9-5.1)	1.4 (0.5-4.1)
GAD	1.5 (0.4-6.1)	2.8 (1.2-6.3)*	1.1 (0.1-13.3)	0.7 (0.1-4.7)
Substance abuse	1.7 (0.5-5.0)	1.5 (0.8-2.7)	0.2 (0.0-2.2)	1.7 (0.4-6.7)
Anti-social personality disorder	1.8 (0.9-3.8)	1.5 (0.9-2.6)	0.8 (0.2-3.0)	0.6 (0.2-2.6)
Suicide (Same-sex parent)	1.5 (0.4-5.0)	3.0 (1.6-5.7)*	0.4 (0.1-1.4)	0.3 (0.0-7.0)
**Number of parental disorders**				
2 or more parental disorders	1.0 (0.2-4.2)	0.6 (0.3-1.5)	3.6 (0.2-53.2)	1.6 (0.2-10.4)

*Significant at the .05 level, two-sided test.

^1^Models control for person-year (1–5 intervals) and demographic factors (age, sex, education level, and marital status).

Parental major depressive disorder, generalized anxiety disorder and suicide were associated with younger age of onset of suicide attempts, as shown in Table 6. A similar though weaker effect was found in the association between offspring suicidal ideation and parental depression, GAD, antisocial personality disorder and suicide. Parental panic disorder was associated with later age of onset of both suicide attempts and ideation in the offspring. Among respondents with suicidal ideation, parental depression was associated with earlier age of onset of suicide plans.

**Table 6:** **Interactions between life course and controls**^**1**^

		**In the total sample**		**Among those with suicidal ideation**	
		**LT attempts**	**Ideators**	**LT plans**	**LT attempts**
**Parental disorder**	**Respondent age**^**2**^	**OR (95% CI)**	**OR (95% CI)**	**OR (95% CI)**	**OR (95% CI)**
Depression	13-19	0.0* (0.0-0.0)*	0.1* (0.0-0.3)*	0.0* (0.0-0.0)*	---
	20-29	0.0* (0.0-0.0)*	0.0* (0.0-0.4)*	0.0* (0.0-0.0)*	---
	30+	0.3 (0.0-3.1)	0.4 (0.0-6.2)	5.1 (0.0-542.1)	---
Panic	13-19	3.0 (0.5-16.4)	3.3* (1.2-8.7)*	0.1 (0.0-1.7)	0.1 (0.0-10.8)
	20-29	6.4* (1.5-27.5)*	2.1 (0.7-6.3)	0.2 (0.0-1.5)	1.0 (0.1-14.8)
	30+	3.8 (0.8-18.4)	2.4 (0.9-6.3)	---	---
GAD	13-19	0.8 (0.1-9.9)	0.2 (0.0-1.4)	0.1 (0.0-22.2)	7.5 (0.0-1395209)
	20-29	0.0* (0.0-0.0)*	0.2* (0.0-0.9)*	0.0 (0.0-15.0)	0.2 (0.0-3.3)
	30+	0.0* (0.0-0.0)*	0.6 (0.1-3.7)	---	---
Substance abuse	13-19	0.4 (0.0-10.0)	1.3 (0.3-5.1)	6.2 (0.0-12221.8)	0.9 (0.0-164695.1)
	20-29	2.6 (0.3-25.9)	0.9 (0.2-3.9)	6.8 (0.0-24235.2)	28.3 (0.0-6400573)
	30+	0.4 (0.0-12.1)	0.3 (0.1-1.8)	---	---
Antisocial personality disorder	13-19	0.9 (0.1-10.8)	0.1* (0.0-0.8)*	---	---
	20-29	1.5 (0.1-20.1)	0.2 (0.0-1.1)	---	---
	30+	0.2 (0.0-5.4)	0.1* (0.0-0.6)*	---	---
Suicide (Same-sex parent)	13-19	0.1 (0.0-13.2)	0.2 (0.0-1.9)	0.0* (0.0-0.0)*	---
	20-29	0.6 (0.1-6.7)	0.3 (0.0-3.0)	0.0* (0.0-0.0)*	---
	30+	0.0* (0.0-0.0)*	0.0* (0.0-0.0)*	0.0* (0.0-0.0)*	---
**Number of parental disorders**					
2+	13-19	12.4 (0.2-781.5)	17.3* (1.5-204.3)*	6.5 (0.0-961.2)	---
	20-29	2.2 (0.1-84.7)	29.0* (2.6-319.9)*	2.4 (0.0-3009.8)	0.5 (0.0-267651.6)
	30+	13.7 (0.2-1031.3)	2.5 (0.1-104.3)	---	---

*Significant at the .05 level, two-sided test.

^1^Models control for person-years and demographic factors, as well as the significant interaction terms between person-years and demographic factors.

^2^Respondent’s age at onset of suicide event.

## Discussion

In this study, a significant proportion of respondents with suicidal ideation reported parental psychopathology. Over a fifth of respondents with suicidal ideation reported at least one parent having a mental illness, with over a quarter of those who attempted suicide reporting parental psychopathology. In a final multivariate model parental panic disorder was significantly associated with respondent suicidal ideation, whereas parental panic disorder, generalized anxiety disorder and suicide maintained a significant association with suicide attempts. Among those with suicidal ideation, none of the tested types of parental psychopathology was associated with suicidal ideation or attempts. There was a pattern consistent with a dose–response relationship between the number of parental disorders and suicidal ideation.

The findings from previous studies, largely in non-African settings, showing that the risk of suicidal ideation is related to parental history of mental illness therefore appear to hold in this study as well [8-10,14]. This is further supported by the finding of a dose–response relationship between parental disorders and suicidal ideation in this study.

Parental psychiatric morbidity is thought to increase risk of suicidal behavior in the offspring through several mechanisms, including the transmission of genetic factors associated with increased levels of distress or dysfunction [38,39], the early environmental stressors that may result from high levels of parental psychopathology [40,41], or a combination of the two [42,43].

Parental panic disorder appears to fit well in this model due to the probability of genetic transmission and the environmental stressors associated with the lifestyle changes instituted by a parent with this disorder. It has for instance been demonstrated that people with panic disorder have significantly increased risk of suicidal behavior [44], and this may be among some of the environmental stressors exerted upon their offspring. Further, Oladeji and Gureje [23] argue that “parental impulsivity and aggressive behavior as is likely to occur in the context of parental panic disorder or substance abuse may predispose to family instability, abuse, and reduced parental care”.

Our findings are to a certain extent similar to those in Oladeji and Gureje’s work in Nigeria [23] which demonstrated an association between parental panic disorder and both suicidal ideation and attempts among offspring. However, our study further found a dose–response relationship with the risk of offspring suicidal behavior increasing with the number of parental mental disorders, a finding that was absent in the Nigerian study perhaps due to the small number of respondents with more than one disorder. Further, unlike the Nigerian study, we found no association between parental substance abuse and offspring suicidal behavior.

Our findings differ in significant ways from those based on the cross-national WMH survey sample, which found that all the tested disorders were significantly associated with respondent suicidal ideation and attempts [18]. For instance, the role of parental depression in increasing the risk of suicidal behavior in the offspring was not replicated in our study. This may be explained in part by the relatively low prevalence of parental depression in our sample compared to the WMHS sample, as well as our lower overall sample size that may have contributed to lower statistical power.

Findings from the cross-national WMHS [18] also suggested that parental depression plays a role in the emergence of suicidal ideation, and that once this emerged, suicide attempts would be mediated by factors including parental antisocial personality and anxiety disorders. The finding in the present study that none of the parental disorders predicts suicidal behavior among those with suicidal ideation suggests that other factors, including potential protective factors, may be relevant in this population. Further research is necessary to uncover these factors and their mechanisms in order to inform relevant interventions.

Among all the types of parental psychopathology examined in this study, only suicide in a parent of the same sex as the respondent predicted respondent suicidal behavior by increasing the risk of offspring suicidal ideation. Previous work has suggested that maternal mental illness and suicide has a greater impact on offspring suicide, and the elevated risk associated with parental psychiatric history was greater in females than in males [22,45]. A recent systematic review and meta-analysis similarly found maternal suicidal behavior to be a more potent risk factor than paternal, and children to be more vulnerable than adolescents and adults [46]. The study however reported no evidence of a stronger association in either male or female offspring.

In contrast with our findings on parental suicide increasing the risk of suicidal behavior in the same-sex offspring, Jeon and colleagues [47] reported that childhood parental death is significantly associated with lifetime suicide attempt in the opposite-sex offspring, especially when exposure occurs before age 10. This finding needs to be further investigated especially in view of the different cultural settings in these two studies.

Our study has several significant limitations that may affect the interpretation of the results. First, errors related to forgetting and recall bias may have been introduced into the results due to the retrospective self-report nature of the instruments used [48]. However, systematic reviews have demonstrated that the level of accuracy in these types of study design is often sufficient to provide useful information [49].

Second, just as in the WMH surveys, this study did not attempt to examine the full range of parental mental disorders, or even of respondent suicidal behaviors that could have been assessed. This limits the findings of this study to the variables that were examined, and it is impossible to demonstrate the effect, if any, of including the other mental disorders and behaviors.

Third, the number of parental disorders in some cases such as depression, generalized anxiety disorder and suicides were relatively low, limiting the ability of this study to provide accurate findings on the association between these disorders and respondent suicidal behavior. A possible reason for this may be the fact that adult offspring were being interviewed about parental psychopathology that may have occurred many years in the past, resulting in under-reporting.

Fourth, it is possible that this study underestimates the associations between the various parental disorders and respondent suicidal behavior because the design did not account for chronicity of the parental disorders. This would suggest that even though many of the findings in this study did not reach statistical significance, they could still be a useful indicator of the magnitude and direction of associations between parental psychopathology and suicidal behavior in their offspring.

Finally, it could be argued that the sample was not sufficiently representative of the South African general population due to the exclusion of those in institutions such as prisons, hospitals, mental hospitals and military barracks. However, due to the weighting of the data and the relatively small proportion of the general population in institutions, we believe that our results adequately reflect the situation in the general population.

## Conclusions

Despite its limitations, this study provides some of the first data from Africa on the relationship between parental psychopathology and suicidal ideation. Significantly, our findings that parental psychopathology is associated with increased risk of suicidal ideation and attempts in offspring warrants further research in this area in order to confirm these findings and possibly elucidate the possible mechanisms.

## Competing interests

The authors declare no competing interests.

## Authors’ contributions

LA was involved in the data analysis and drafted the manuscript. MKN was involved in the study design and data collection and analysis. DRW was involved in the study design and data collection and analysis. DJS was involved study design, data collection and analysis and helped in the preparation of the draft manuscript. All authors read and approved the final version of the manuscript.

## Pre-publication history

The pre-publication history for this paper can be accessed here:

http://www.biomedcentral.com/1471-244X/14/65/prepub
